# Clinical and paraclinical profile, and predictors of outcome in 90 cases of scrub typhus, Meghalaya, India

**DOI:** 10.1186/s40249-016-0186-x

**Published:** 2016-10-05

**Authors:** Sunuraj Sivarajan, Siddharudha Shivalli, Debomallya Bhuyan, Michael Mawlong, Rittwick Barman

**Affiliations:** 1 Department of General Medicine, Nazareth Hospital, Shillong, 793003 Meghalaya India; 2 Department of Community Medicine, Yenepoya Medical College, Yenepoya University, Mangalore, 575018 Karnataka India; 3 Department of Microbiology, Nazareth Hospital, Shillong, 793003 Meghalaya India

**Keywords:** Hospital, India, Longitudinal, Multiple Organ Dysfunction Syndrome (MODS), Mortality, Scrub typhus

## Abstract

**Background:**

India is an integral component of “*tsutsugamushi* triangle” which depicts a part of the globe endemic to scrub typhus. Owing to frequent outbreaks witnessed in different parts of the country in the recent past, scrub typhus is described as a re-emerging infectious disease in India. The present study aimed to study the clinical and paraclinical profile, complications and predictors of outcome among 90 cases of scrub typhus diagnosed in a hospital of north-eastern India from Sept 2011 to Aug 2012.

**Methods:**

A longitudinal study was conducted in a hospital of Meghalaya, India between Sept 2011 and Aug 2012. Diagnosis of scrub typhus was arrived by SD BIOLINE tsutsugamushi (solid phase immunochromatographic assay) rapid diagnostic test for antibodies (IgM, IgG or IgA). Descriptive analyses of age, gender, geographic area, symptoms and signs, treatment, laboratory findings, complications, and outcome were conducted. Relative risk (RR) with 95 % confidence interval (*CI*) was computed for Multiple Organ Dysfunction Syndrome (MODS) and mortality. Binary logistic regression was applied to the significant correlates (*P* < 0.05) on univariate analysis to identify the predictors of MODS and mortality in scrub typhus.

**Results:**

As many as 662 clinically suspected scrub typhus patients were tested and 90 (13.6 %) were diagnosed to have scrub typhus. Out of 90 patients, 52.2 % (*n* = 47) were males and their mean (SD) age was 36.29 (13.38) years. Fever of <7 days (*n* = 75, 83.3 %), myalgia (*n* = 56, 62.2 %), pain abdomen (*n* = 24, 26.7 %), headache (*n* = 24, 26.7 %), nausea/vomiting (*n* = 21, 23.3 %), dry cough (*n* = 21, 23.3 %), hepatomegaly (*n* = 24, 26.7 %), splenomegaly (*n* = 22, 24.4 %), and lymphadenopathy (*n* = 20, 22.2 %) were the predominant clinical features. Eschar was seen in 10 patients (11.1 %). One third (*n* = 30) of the patients developed at least one systemic complication. Acute hepatitis (*n* = 15, 16.7 %), pneumonitis (*n* = 14, 15.6 %), and acute kidney injury (*n* = 11, 12.2 %) were the common complications. MODS was seen in 14.4 % (*n* = 13) and 38.5 % (*n* = 5) of the patients with MODS died. Overall, case fatality rate was 5.15 % (*n* = 5). On univariate analysis, platelets <100 000/mm^3^, serum creatinine >1.5 mg/dl, and transaminase (AST, ALT or both) >500 U/L were associated with MODS (*P* < 0.001) and mortality (*P* < 0.05). In addition, serum bilirubin >3 mg/dl was also associated with MODS (*P* < 0.001). On applying binary logistic regression, serum creatinine >1.5 mg/dl was a predictor of MODS (*OR*: 76.1, 95 % *CI*: 4.9–1175.6) and mortality (*OR*: 18.03, 95 % *CI*: 1.38–235.1).

**Conclusion:**

In this study setting, approximately one-seventh (13.6 %) of the acute undifferentiated febrile illness were due to scrub typhus. Systemic complications were common (33.3 %). Serum creatinine >1.5 mg/dl was a predictor of MODS and mortality.

**Electronic supplementary material:**

The online version of this article (doi:10.1186/s40249-016-0186-x) contains supplementary material, which is available to authorized users.

## Multilingual abstracts

Please see Additional file 1 for translation of the abstract into the six official working languages of the United Nations.

## Background

Scrub typhus is a vector-borne zoonotic disease caused by Gram-negative bacterium *Orientia tsutsugamushi*. It is transmitted to humans through the bite of larval trombiculid mite [1, 2]. The infection is maintained in nature transovarially from one generation of mite to the next. Humans are the accidental dead end hosts. After 9–12 days, a typical skin lesion known as eschar is formed at the mite bitten site. It is an acute febrile illness with nonspecific clinical features like high fever, maculopapular rash, lymphadenopathy, headache, and myalgia.

India is an integral component of “*tsutsugamushi* triangle” which depicts a part of the globe endemic to scrub typhus. The “*tsutsugamushi* triangle” extends from northern Japan and far-eastern Russia in the north, to northern Australia in the south, and to Pakistan in the west [3]. Scrub typhus is one of the important causes of acute undifferentiated febrile illnesses in Asia [4]. High index of suspicion and careful examination for eschar at the bite site is vital for the clinical diagnosis. States like Assam and West Bengal reported the first scrub typhus epidemics in India during World War II. Later scrub typhus was reported in humans and experimental animals exposed in these areas [5]. Owing to frequent outbreaks witnessed in different parts of the country in the recent past, scrub typhus is described as a re-emerging infectious disease in India [4, 6–9]. The World Health Organization (WHO) describes scrub typhus as one of the most under diagnosed and underreported febrile illnesses requiring hospitalization. It strongly emphasizes surveillance owing to its relatively high case fatality rate (up to 30 % in untreated patients) and severe underreporting [10].

The present study aimed to describe the clinical and paraclinical profile, complications and predictors of outcome among 90 cases of scrub typhus in a hospital of north-eastern India from Sept 2011 to Aug 2012.

## Methods

### Study setting and design

A longitudinal study was conducted from September 2011 to August 2012 in a hospital, Shillong, Meghalaya, India. This is a 375-bedded multi-specialty hospital in Shillong, the capital of Meghalaya in East Khasi Hills district. The Medicine inpatient facilities include 100 beds.

Meghalaya is one of the seven sister states of northeast India with heavy rainfall and agriculture is the main source of economy. East Khasi Hills District is located in the central part of the Meghalaya and covers a total geographical area of 2 748 Sq. Kms. It lies approximately between 25°07” & 25°41” N Lat. and 91°21” & 92°09” E Long [11]. The East Khasi Hills is a typical hilly district with deep gorges and ravines on the southern part. The climate of the district ranges from temperate in the plateau region to warmer tropical and sub-tropical pockets on the Northern and Southern regions. The weather is humid for the major part of the year except for the relatively dry spell usually between December and March [11].

### Case definition

- Suspected case
  Acute undifferentiated febrile illness of ≥5 days with/without eschar.
- Confirmed case
  A suspected case of scrub typhus was confirmed by SD Bioline Tsutsugamushi (Standard Diagnostics, Inc., Gyonggi-do, Korea) rapid diagnostic test for IgG, IgM or IgA antibodies [12] and a dramatic response to doxycycline. SD Bioline Tsutsugamushi test is a solid phase immune-chromatographic assay for the rapid, qualitative detection of IgG, IgM or IgA antibodies to *Orientia tsutsugamushi* in human serum, plasma or whole blood. It has a high sensitivity (99 %), specificity (96 %) and serological agreement (97.5 %) with immunofluorescent assay [13]. A correlation of 97 %, between IgM ELISA and SD Bioline Tsutsugamushi rapid diagnostic test, was reported by Ramyasree A et al. [14] among 100 suspected cases of scrub typhus in India.

All consecutive patients, aged 18 years and above, presenting with febrile illness were evaluated. Detailed clinical examination, including a careful search for eschar was made in all the patients. All of them were evaluated for other endemic febrile diseases, i.e., malaria, typhoid fever, dengue, leptospirosis, and pneumonia by relevant laboratory tests. Basic laboratory tests like complete blood count, renal function tests (blood urea and serum creatinine), blood glucose, and liver function tests [serum bilirubin (direct and indirect), aspartate aminotransferase (AST), alanine aminotransferase (ALT) and alkaline phosphatase (ALP) and serum albumin] were done. Other investigations, including chest X-ray, Widal test, blood culture, cerebrospinal fluid (CSF) analysis, and ultrasonography of abdomen were done as indicated.

The following criteria were used to define various systemic complications in scrub typhus [15].

1. Acute Kidney Injury (AKI): Rise of serum creatinine (Scr) of at least 0.3 mg/dl or 50 % higher than baseline within a 24–48-h period or a reduction in urine output to 0.5 mL/kg per hour for longer than 6 h.
2. Acute hepatitis: Elevation of serum transaminases more than 6 times the normal upper limit.
3. Acute respiratory distress syndrome (ARDS): Acute onset of non-cardiogenic pulmonary edema manifesting with bilateral alveolar or interstitial infiltrates on chest radiograph and PaO_2_/FIO_2_ ≤ 200 mmHg on arterial blood gas analysis.
4. Pneumonitis: Acute onset of non-cardiogenic pulmonary edema manifesting with unilateral or bilateral alveolar or interstitial infiltrates on chest radiograph and PaO_2_/FIO_2_ > 200 mmHg on arterial blood gas analysis.
5. Disseminated intravascular coagulation (DIC): Prolongation of PT and/or aPTT; platelet counts 100 000/μl^3^, or a rapid decline in platelet numbers over 24 h; the presence of schistocytes (fragmented red cells) in the blood smear; and elevated levels of fibrin degradation products (FDPs).
6. Pancreatitis: Acute onset of clinical symptoms such as abdominal pain, vomiting, guarding/ tenderness associated with elevation of serum amylase/ lipase >3 times upper limit of normal.
7. Septic shock: Systolic blood pressure of <90 mmHg for at least 1 h despite adequate fluid resuscitation.
8. Meningitis: Altered sensorium and signs of meningeal irritation associated with elevated protein and lymphocytic/neutrophilic cytology with normal or low sugar on CSF analysis.
9. Multiple Organ Dysfunction Syndrome (MODS): Dysfunction of more than one organ, requiring intervention to maintain homeostasis.

Confirmed case of scrub typhus was given doxycycline in the dosage of 100 mg twice a day for 10 days. All other supportive measures such as haemodialysis, ventilator support, transfusion of blood components, and inotropic support were given as per the indications.

### Statistical analysis

Data were analyzed using Statistical Package for the Social Sciences (SPSS) Inc, Chicago, USA; Version 16.0. Continuous variables were expressed in mean and standard deviations (SD) or median and inter quartile range (IQR). Categorical variables were expressed as number and percentages. Study variables considered in the descriptive analysis were: gender, age, district of origin, symptom profile on admission, presence of co-morbidities, physical examination on admission, initial laboratory findings, incident complications, and outcome of the treatment. Relative risk (RR) with 95 % confidence interval (CI) was computed for MODS and mortality. Binary logistic regression was applied to the significant correlates (*P* <0.05) on univariate analysis to identify the predictors of MODS and mortality in scrub typhus. A two sided *P* < 0.05 was considered as statistically significant.

## Results

### Demographic profile

As many as 662 clinically suspected scrub typhus patients were tested by SD Bioline *Tsutsugamushi* during the study period from September 2011 to August 2012. A total of 90 (13.6 %) patients were diagnosed to have scrub typhus and all of them completed the study. Maximum numbers of cases were seen during the cooler (temperature range: 0–17 °C) months between September and December. Forty seven out of 90 (52.2 %) patients were males and their mean age (SD) was 36.3 (13.4) years. Forty one out of 90 (45.6 %) belonged to the age group of 21–30 years. Patients from nearby four districts presented to the study hospital and 54.4 % (*n* = 49) were from the East Khasi Hill district [Table 1]. Among the 90 patients, farmers (*n* = 19, 21.1 %), labourers (*n* = 17, 18.9 %), homemakers (*n* = 14, 15.6 %), and unemployed (*n* = 16, 17.8 %) constituted 73.4 % (*n* = 66) [Table 1].

**Table 1:** Key socio-demographic parameters of the scrub typhus patients in a tertiary care hospital of Meghalaya, India, Sept 2011–Aug 2012 (*n* = 90)

Study variable	*n*	%
Age group (years)		
18–20	4	4.4
21–30	41	45.6
31–40	17	18.9
41–50	15	16.7
51–60	7	7.8
>60	6	6.7
Gender		
Male	47	52.2
Female	43	47.8
Geographic location (District)		
East Khasi Hills	49	54.4
Jaintia Hills	21	23.3
West Khasi Hills	17	18.9
Ribhoi	3	3.3
Occupation		
Farmer	19	21.1
Labourer	17	18.9
Home maker	14	15.6
Businessman	8	8.8
Student	4	4.4
Others	8	8.8
Unemployed	16	17.8
Retired	4	4.4

### Clinical features

**Fig. 1:**
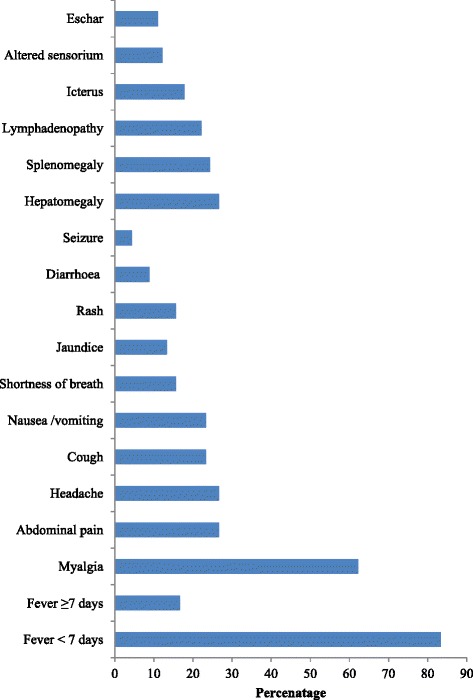
Clinical symptoms and signs among hospitalized patients with scrub typhus Meghalaya, India, Sept 2011–Aug 2012 (*n* = 90)

Fever of <7 days (*n* = 75, 83.3 %), myalgia (*n* = 56, 62.2 %), pain abdomen (*n* = 24, 26.7 %), headache (*n* = 24, 26.7 %), nausea/vomiting (*n* = 21, 23.3 %), and dry cough (*n* = 21, 23.3 %) were the predominant symptoms among scrub typhus patients [Fig. 1]. Most of them had high grade intermittent fever. Myalgia, although not assessed objectively, was not as prominent as seen in dengue and leptospirosis. Abdominal pain (*n* = 24, 26.7 %) and headache (*n* = 24, 26.7 %) were very prominent symptoms in some of the patients.

Hepatomegaly (*n* = 24, 26.7 %), splenomegaly (*n* = 22, 24.4 %), and lymphadenopathy (*n* = 20, 22.2 %) were the predominant clinical signs. Out of 11 patients (12.2 %) who had altered sensoruim, two were diagnosed to have meningitis. Eschar was found only in 11.1 % (*n* = 10) of the patients. Some patients who complained of breathing difficulty and in whom chest X-ray showed infiltrates were managed conservatively in the ward with doxycycline, fluid restriction, and low dose diuretics. However, those who developed ARDS were managed in Intensive Care Unit (ICU) and mechanically ventilated. Other signs like icterus (*n* = 16) and maculopapular rashes (*n* = 14) were seen in 17.8 and 15.6 %, respectively.

### Laboratory findings

Almost all of them displayed raised liver enzymes in the serum, i.e. AST, ALT and ALP. As much as 63.3 % of them (*n* = 57) had thrombocytopenia and one third (*n* = 18, 20 %) of these had a platelet count <100 000 per μl. However, 62.3 % (*n* = 56) of them had normal leucocyte count. One fourth of them had hyperbilirubinemia, leucocytosis, and hypoalbunemia [Table 2]. Leucopenia, including agranulocytosis, was seen in 12 % (*n* = 11) of the cases. Patients with agranulocytosis were managed with broad spectrum antibiotics apart from doxycycline.

**Table 2:** Laboratory findings of patients with scrub typhus in a tertiary care hospital of Meghalaya, India, Sept 2011–Aug 2012 (*n* = 90)

Laboratory parameter	*N*	%
Leucocytosis (>12 000/μl)	23	25
Leucopoenia (<4 000/μl)	11	12
Thrombocytopenia (per μl)		
<165 000	57	63.3
<100 000	18	20
Serum bilirubin >1.3 mg/dl	23	25
Aspartate aminotransferase >40 IU	90	100
Alanine aminotransferase >40 IU	85	94
Alkaline phosphatase >130 IU/L	74	82
Serum creatinine >1.6 mg/dl	13	14
Serum albumin < 3 g%	21	23

Incidentally, some unique abnormalities were seen in 11 patients with jaundice. Most of these patients were characteristically found to have conjugated bilirubinemia with elevated liver enzymes [Additional file 2]. AST (median: 460 IU/L, IQR: 207–627) values were found to be consistently higher than ALT values (median: 102 IU/L, IQR: 86–109.5); the ratio of AST/ALT was >1. ALP was markedly elevated in the majority of these patients. None of the patients showed any alteration of hepatic echo texture or obstructive features in ultrasonography.

### Complications

One third (*n* = 30) of the patients developed at least one systemic complication. Acute hepatitis (*n* = 15, 16.7 %), pneumonitis (*n* = 14, 15.6 %), and AKI (*n* = 11, 12.2 %) were the common complications. Multisystem involvement was seen in 14.4 % (*n* = 13) of them. Shock requiring inotropes was seen in two patients and both succumbed to their illness. Six others had hypotension responsive to fluids. Five patients developed meningitis.

AKI and acute hepatits were seen in most of the patients with multisystem involvement [Table 3]. Five patients who presented with breathing difficulty developed ARDS. Two of them recovered after mechanical ventilation while three died. Five out of 13 patients with MODS (38.5 %) died and the overall case fatality rate was 5.15 %. All the five deaths were among women.

**Table 3:** Salient features of patients with scrub typhus and Multiple Organ Dysfunction Syndrome (MODS) in a hospital of Meghalaya, India, Sept 2011–Aug 2012 (*n* = 13)

No	Age (years)	Sex	TC^†^	Plt/mm^3††^	Hypotension/shock	ARDS^§^	Meningitis	Acute hepatitis	AKI^¶^	Outcome
1	24	M	15 800	160 000	Hypotension	✓		✓	✓	Recovered
2	24	M	16 200	45,000			✓	✓		Recovered
3	25	M	12 500	19 000	✓			✓		Recovered
4	25	M	13 300	141 000				✓	✓	Recovered
5	25	F	20 900	120 000		✓	✓			Expired
6	26	M	4 600	16 200				✓	✓	Recovered
7	27	M	15 700	24 000				✓	✓	Recovered
8	29	F	15 100	28 000	Hypotension	✓		✓	✓	Expired
9	42	F	37 000	215 000	✓	✓			✓	Expired
10	44	F	10 600	27 000			✓	✓	✓	Expired
11	46	F	12 000	72 000				✓	✓	Recovered
12	70	F	13 300	80 000	✓			✓	✓	Expired
13	76	F	16 200	45 000			✓	✓	✓	Recovered

^†^ *TC* total count, ^††^ *Plt/mm* ^*3*^ platelets/cubic millimeter, ^§^ *ARDS* Acute Respiratory Distress Syndrome, ^¶^ *AKI* Acute Kidney Injury

Lymphocytic leukocytosis was the typical feature on CSF examination of those with meningitis [Table 4]. Median CSF cell count was 101/cmm (IQR: 91–111). Protein was elevated in all these patients (median: 122 mg/dl, IQR: 113–143). CSF sugar was low in two patients (median: 25 mg/dl, IQR: 25–56). Adenosine deaminase was ≥10 U/L in two patients (median: 8.8 U/L, IQR: 7.8–10).

**Table 4:** Cerebrospinal fluid (CSF) findings among patients with scrub typhus meningitis in a tertiary care hospital of Meghalaya, India, Sept 2011–Aug 2012 (*n* = 5)

No	Age (years)	Sex	Total count (/mm^3^)	Polymorphs (%)	Lymphocytes (%)	Protein (mg/dl)	Sugar (mg/dl)	RBC (mm^3^)^a^	ADA^b (U/L)^
1	24	Male	220	1	99	122	56	50	5.0
2	25	Female	91	2	89	155	25	–	7.8
3	44	Female	101	9	89	93	17	30	10.0
4	53	Female	11	3	8	143	25	–	11.0
5	76	Female	111	4	96	113	63	–	8.8

^a^ *RBC* red blood cell, ^b^ *ADA* adenosine deaminase

### Co-morbidities

Co-morbidities were seen in two patients; one was a pregnant woman and the other had malaria co-infection. The pregnant woman with scrub typhus was 19 years old with a gestational age of 16 weeks. She presented with acute onset of fever, myalgia and no specific signs on examination. Leucocytosis and elevated liver enzymes were the only significant laboratory findings. She responded well with azithromycin treatment with no incident complications. However, she did not come to the study hospital for delivery and therefore we could not assess the obstetric outcome.

Another 25 year old male patient presented with shock, icterus, and hepatospenomagaly and on investigation he had *Plasmodium falciparum* co-infection, severe thrombocytopenia (19 000/μl) and acute hepatitis. In addition to doxycycline, he was treated with artesunate combination therapy as per the national malaria treatment guidelines [16]. The patient responded to transfusion of blood components and inotropic support.

### Predictors of MODS and mortality

On univariate analysis, platelets <100 000/mm^3^, serum creatinine >1.5 mg/dl, and transaminase (AST, ALT or both) >500 U/L on admission were associated with MODS (*P* < 0.001) and mortality (*P* <0.05) [Table 5]. In addition, serum bilirubin >3 mg/dl (*RR*: 16.16, 95 % *CI*: 5.97–43.7) was also associated with MODS (*P* < 0.001). On applying binary logistic regression, serum creatinine >1.5 mg/dl was a predictor of MODS (*OR*: 76.1, 95 % *CI*: 4.9–1175.6) and mortality (*OR*: 18.03, 95 % *CI*: 1.38–235.1).

**Table 5:** Relative risks (RR) for Multiple Organ Dysfunction Syndrome (MODS) and mortality among patients with scrub typhus in a hospital of Meghalaya, India, Sept 2011–Aug 2012 (*n* = 90)

Study variable	MODS (*n* = 13)			Mortality (*n* = 5)		
	*RR*	95 % *CI*	*p*	*RR*	95 % *CI*	*p*
Gender (female vs. male)	1.3	0.46–3.5	0.637	12	0.68–210.8	0.637
Presence of eschar	2.4	0.79–7.28	0.122	2.0	0.25–16.17	0.515
Fever (<7 days vs. >7 days)	2.4	0.34–17.1	0.38	2.32	0.14–39.8	0.562
Occupation (farmer / labourer vs. others)	0.45	0.13–1.52	0.199	1.0	0.17–5.69	1.00
Age (≥60 years vs. <60 years)	1.63	0.43–6.24	0.471	2.25	0.2–18.02	0.44
Serum Bilirubin >3 mg/dl	16.16	5.97–43.7	<0.001^*^	4.78	0.89–25.54	0.066
Platelet count <100,000/mm^3^	9.0	3.12–25.9	<0.001^*^	6.0	1.082–33.2	0.04^*^
Serum creatinine >1.5 mg/dl	29.9	7.4–120.4	<0.001^*^	21.7	2.6–180.2	0.004^*^
AST, ALT or both >500 IU/L^†^	7.7	3.3–17.9	<0.001^*^	6.0	1.15–31.2	0.03^*^

*Abbreviations*: *MODS* Multiple Organ Dysfunction Syndrome, *RR* relative risk, *CI* confidence interval, ^†^ *AST* Aspartate aminotransferase, *ALT* Alanine aminotransferase; ^*^statistically significant (*P* < 0.05)

The ARDS taskforce published the Berlin definition of ARDS in June 2012 [17]. This study was started in Sept 2011 and hence, we did not use these criteria for ARDS. According Berlin definition, pneumonitis and ARDS used in this study are classified as mild and moderate ARDS, respectively [17]. However, even with the Berlin definition of ARDS, serum creatinine >1.5 mg/dl was a predictor of MODS (*OR*: 35.32, 95 % *CI*: 4.2–299.7) and mortality (*OR*: 50.3, 95 % *CI*: 3.4–734.8).

## Discussion

### Key findings

In this study setting, 13.6 % of acute undifferentiated febrile illness was attributed to scrub typhus. One third (*n* = 30) of the patients developed at least one systemic complication. MODS was seen in 14.4 % (*n* = 13) and 38.5 % of patients with MODS died, and the overall case fatality rate was 5.15 %. Serum creatinine >1.5 mg/dl was a predictor of MODS (*OR*: 76.1, 95 % *CI*: 4.9–1175.6) and mortality (*OR*: 18.03, 95 % *CI*: 1.38–235.1).

### Interpretation

Although many states of India have reported the disease outbreak, paucity of data hinders further research on scrub typhus. This longitudinal study was an attempt to explore the predictors of complications and outcome in scrub typhus. Corroborating with other studies [6, 10, 15, 18–22], the present one also reaffirms that scrub typhus commonly presents with non-specific symptoms like acute onset of fever with myalgia, breathlessness, cough, nausea, vomiting, headache etc. It is difficult to differentiate scrub typhus from other co-endemic diseases like malaria, dengue, and leptospirosis. Therefore, a high index of clinical suspicion, exploring the history of environmental exposure, and vigilant search for the eschar are crucial for diagnosis.

Seasonal occurrence of scrub typhus is seen and it varies with the climate in different countries. Epidemic period is influenced by the activities of the infected mite and often occurs during the rainy season [23, 24]. However, similar to our study, outbreaks have been reported during the cooler season or post monsoon, in India [9, 25–27]. In the cooler months, there is an increase in secondary shrub vegetation which in turn favors the growth of the vector. In the same season, farmers are involved in harvesting activity in the fields, where they are exposed to the bites of larval mites [28, 29]. Therefore, intensified health education activities are needed in the rainy and post monsoon sessions to cut down the transmission. Targeted preventive interventions like personal protection are to be canvassed among the high risk groups like farmers and those involved in collecting firewood from jungle.

This study and other studies conducted in India [5, 9] and Asia [19, 20] have shown lower positivity for the eschar (8–15 %) in scrub typhus. Indigenous patients of typhus endemic areas regularly tend to have less severe illness, often without rash or eschar [21]. Whether this is due to past exposure to the organism, variation in strain type or other factors needs to be explored. Therefore, in the Indian context absence of eschar does not rule out the diagnosis.

As observed in our study and by others [9, 22, 30] elevated serum tarnsaminase levels and thrombocytopenia appears to be a consistent paraclinical feature in scrub typhus. In a study by *Varghese GM* et al. (*n* = 50), a combination of elevated transaminases, thrombocytopenia and leukocytosis displayed 80 % specificity and positive predictive value for scrub typhus diagnosis [9]. This could be very useful to primary care physicians who may not have immediate access to confirmatory tests. However, this association needs to be further validated by analytical epidemiological studies on larger samples.

The present study like some others [27, 30–32] suggest that systemic complications are common in scrub typhus. Both agent and the host factors are decisive for the occurrence of complications. *Orientia tsutsugamushi* has more than 20 antigenically distinct regionally distributed serotypes and some strains seem to have higher virulence [31]. Host factors like older age, co-morbidities, paraclinical features on admission and delayed onset of treatment seem to contribute to the disease severity and complications. Occurrence of systemic complications like shock, acute renal failure, meningitis, and MODS are associated with a higher mortality [32]. A wide range of case fatality rate for scrub typhus (5–30 %) is reported in India [4, 9, 32, 33] and across the globe [21, 34]. However, a decreasing trend in the mortality is evident over the years [32, 33]. It could be attributed to increased awareness, early seeking of healthcare, timely initiation of antibiotic treatment and/or possible lower virulent strain of *tsutsugamushi* in the area.

In the present study, gender wise (women vs. men) differences in MODS (16.3 % vs. 12.8 %) and fatality rate (11.6 % vs. 0 %) were statistically not significant (*P* > 0.05). Whether it was a serendipitous finding or due to biological susceptibility or social factors such as ignorance and delay in seeking care, need to be investigated. Similar to our finding, creatinine >1/4 mg/dl was an independent predictor of mortality in scrub typhus in a study from southern India [8]. Fever of >12 days, presence of eschar, ICU admission, shock needing ionotropes, CNS dysfunction etc. have also been proven to be predictors of mortality [5, 14, 35]. These give valuable hints to the impending fatal outcome and may help the clinicians to initiate the intensive management.

Although doxycycline is the drug of choice for scrub typhus [36, 37], clinical failure and resistance is reported [38, 39]. A meta-analysis by *Panpanich R* et al. [40] reveals the paucity of high quality evidence about the antibiotics for scrub typhus. However, the existing evidence suggests that there is no obvious differences between tetracycline, doxycycline, telithromycin, or azithromycin for effectiveness and rifampicin may be preferred when response to standard anti‐rickettsial drugs is poor [40]. Randomized controlled trials are needed to generate higher quality evidence.

Although scrub typhus is rare in pregnancy, it needs a special mention owing to possible unfavorable pregnancy outcome. Clinical features of scrub typhus among pregnant and non-pregnant women appear to be similar. A wide range of pregnancy outcome such as abortion, still birth, low birth weight, maternal and neonatal deaths have been reported [41–45]. However, available evidence is insufficient to conclude about the effect of scrub typhus on pregnancy outcome [46]. Although, azithromycin is the preferred drug in pregnancy, prospective studies are needed to generate a higher level of evidence [46].

Behavioral risk factors such as outdoor activity or sleeping, lack of personal protective measures and conducive environment for the vector, are common for the vector borne co-endemic diseases like scrub typhus, malaria, leptospirosis and dengue in India. Therefore, co-infections are not uncommon [47–52]. Early identification of such co infections is crucial as their treatments differ drastically and also to avert the complications and mortality. However, overlapping clinical and paraclinical features often challenge the clear-cut distinction from each other. Concurrent confirmatory testing of these diseases is needed, especially if patient does not respond to doxycycline. Nonetheless, the diagnostic yield and cost effectiveness of the same need to be evaluated.

Central nervous system involvement is not uncommon in scrub typhus with non-specific CSF and neuro-radiology findings [53]. In this study, CSF findings in scrub typhus meningitis mimicked tuberculosis except for ADA levels (two had ADA ≥10 U/L). Similar findings are reported by *Viswanathan S* et al. in a retrospective study from south India [54]. Unless, specific tests are employed, scrub typhus meningitis may go undetected may be wrongly treated for tubercular meningitis. Considering the non-availability of confirmatory tests at all levels of healthcare, CSF ADA may be useful in differentiating scrub typhus and tubercular meningitis. However, further studies are needed in this regard [54].

This study had the following limitations: Hospital based study population may not represent the scrub typhus burden in the community. This study relied on rapid test for confirmation due to the cost and lack of wider availability of confirmatory tests. However, a correlation of 97 %, between IgM ELISA and SD Bioline Tsutsugamushi rapid test was reported in India [14] and it was more sensitive than standard IFA in acute phase from Thailand [55]. Moreover, a dramatic response to doxycycline strongly favors the scrub typhus diagnosis. Nevertheless, further studies are needed to find the most suitable test for scrub typhus in terms of the accuracy, rapidity, simplicity of the procedure, ease of interpretation and cost to be used in the Indian populace [56].

## Conclusion

In this study setting, approximately one-seventh (13.6 %) of the acute undifferentiated febrile illness were attributed to scrub typhus. Systemic complications were common (33.3 %), and MODS was associated with higher mortality (38.5 %). Serum creatinine gives valuable hints to the impending fatal outcome and helps the clinicians to initiate the intensive management. The diagnostic yield and cost-effectiveness of concurrent testing for scrub typhus and other co-endemic diseases among acute febrile patients in endemic settings, need to be evaluated.

## Additional files

Additional file 1: Multilingual abstract in the six official working languages of the United Nations. (PDF 770 kb)
Additional file 2: Findings of liver function test among patients with scrub typhus and serum bilirubin >3 mg/dl in a tertiary care hospital of Meghalaya, India, Sept 2011–Aug 2012 (*n* = 11). (DOCX 13 kb)
Additional file 3: Data spreadsheet. (XLSX 35 kb)

## Abbreviations

AKI: Acute Kidney Injury
ALP: Alkaline phosphatase
ALT: Alanine aminotransferase
ARDS: Acute respiratory distress syndrome
AST: Aspartate aminotransferase (AST)
CSF: Cerebrospinal fluid
DIC: Disseminated intravascular coagulation
IQR: Inter Quartile range
MODS: Multiple Organ Dysfunction Syndrome
SPSS: Statistical Package for the Social Sciences
WHO: World Health Organization
