# Supplementary material for: Clinical and paraclinical profile, and predictors of outcome in 90 cases of scrub typhus, Meghalaya, India
Source: Infect Dis Poverty. 2016 Oct 5;5:91. doi: 10.1186/s40249-016-0186-x (PMC5051022; doi:10.1186/s40249-016-0186-x)
Supplement: Additional file 1: — Multilingual abstract in the six official working languages of the United Nations. (PDF 770 kb) [file 40249_2016_186_MOESM1_ESM.pdf]

Translation of the abstract into the six official working languages of the United Nations

دراسة سريرية وشبه سريرية ، ومؤشرات للنتائج في 90 حالة من التيفوس الحكي، ميجالايا، الهند

سونوراج سيفاراجان، سيدارودا شيفلي، ديبوماليا بهويان، مايكل ماولونج، ريتويك بارمان

ملخص

خلفية: الهند هي جزء لا يتجزأ من "مثلث تسوتسوجاموشي" الذي يشتمل على جزء من العالم يتوطن فيه التيفوس الحكي. ونظرا للتفشي المتكرر للمرض الذي شهدته مناطق مختلفة من البلاد في الماضي القريب، يوصف التيفوس الحكي كمرض معد متكرر الظهور في الهند. هدفت الدراسة الحالية إلى إجراء دراسة سريرية وشبه السريرية ، ودراسة المضاعفات والتنبؤ بالنتائج بين 90 حالات تيفوس حكي تم تشخيصها في مستشفى في شمال شرق الهند من سبتمبر 2011-أغسطس 2012.

الطرق: أجريت دراسة طولية في مستشفى في ميجالايا، الهند بين سبتمبر 2011 وأغسطس 2012. تم تشخيص التيفوس الحكي من خلال اختبار التشخيص السريع للأجسام المضادة SD BIOLINE تسوتسوجاموشي (المرحلة الصلبة في الفحص المناعي) (IgM أو IgG أو IgA). أجريت التحليلات الوصفية للعمر، والجنس، والمنطقة الجغرافية، والأعراض والعلامات والعلاج والنتائج المخبرية، والمضاعفات، والنتيجة. تم حساب الاختطار النسبي (RR) مع فاصل الثقة 95٪ (CI) لمتلازمة قصور الأعضاء المتعدد والوفيات. تم تطبيق الانحدار اللوجستي الثنائي ليرتبط معنويا (P <0.05) على تحليل وحيد المتغير لتحديد منبئات متلازمة قصور الأعضاء المتعدد والوفيات في التيفوس الحكي.

النتائج: تم اختبار ما يصل إلى 662 يشتبه سريريا بإصابتهم بالتيفوس الحكي وتم تشخيص 90 حالة (13.6٪) مصابة بالتيفوس الحكي. من 90 مريضا، كانت 52.2٪ (n = 47) ذكور بمتوسط سن (SD) 36.29 (13.38) سنة. المظاهر السريرية التالية كانت سائدة وتشمل حمى >7 أيام (n = 75، 83.3٪)، ألم عضلي (n = 56، 62.2٪)، ألم بالبطن (n = 24، 26.7٪)، صداع (ن = 24، 26.7٪)، غثيان/قيء (n = 21، 23.3٪)، سعال جاف (n = 21، 23.3٪)، تضخم الكبد (n = 24، 26.7٪)، تضخم الطحال (n = 22، 24.4٪) اعتلال العقد اللمفية (n = 20، 22.2٪). لوحظ خُشَارَةٌ (تقشر الجلد) في 10 مرضى (11.1٪). ثلث المرضى (n = 30) عانى من إحدى المضاعفات النظامية على الأقل. التهاب الكبد الحاد (n = 15، 16.7٪)، التهاب رئوي (n = 14، 15.6٪)، وقصور كلوي حاد (n = 11، 12.2٪) كانت مضاعفات شائعة. ظهرت متلازمة قصور الأعضاء المتعدد في 14.4٪ (n = 13) وتوفي 38.5٪ (n = 5) من المرضى الذين يعانون من متلازمة قصور الأعضاء المتعدد. وعموما، كان معدل إماتة الحالات 5.15٪ (n = 5). ووفقا للتحليل وحيد المتغير، كانت الصفائح الدموية <100 000/مم مكعب، الكرياتينين> 1.5 مجم/ دل وناقلة (AST، ALT أو كليهما)> 500 U / L مرتبطة بمتلازمة قصور الأعضاء المتعدد (P <0.001) والوفيات (P <0.05). وبالإضافة إلى ذلك، ارتبط البيليروبين في الدم> 3 مجم/ دل أيضا بمتلازمة قصور الأعضاء المتعدد (P <0.001). وبتطبيق الانحدار اللوجستي الثنائي، كان مصل الكرياتينين> 1.5 مجم/ دل منبئ بالإصابة بمتلازمة قصور الأعضاء المتعدد (OR: 76.1، 95٪ CI: 4.9-1 175.6) والوفيات (OR: 18.03، 95٪ CI: 1.38-235.1).

الخلاصة: في هذه الدراسة، كان السبع تقريبا (13.6٪) من مرض الحمى غير المتمايزة الحادة يرجع إلى التيفوس الحكي. كانت المضاعفات الجهازية شائعة (33.3٪). كان مصل الكرياتينين> 1.5 مجم/ دل مؤشرا لمتلازمة قصور الأعضاء المتعدد والوفيات.

Translated from English version into Arabic by Mahmoud Sami, through

TRANSLATORS WITHOUT BORDERS

印度梅加拉亚邦 **90** 例恙虫病患者的临床和辅助临床资料及疗效预测指标

Sunuraj Sivarajan, Siddharudha Shivalli, Debomallya Bhuyan, Michael Mawlong, Rittwick Barman

摘要

引言：印度是“恙虫病大三角”不可或缺的组成部分，恙虫病分布在一个日本北部、澳大利亚西部和俄罗斯中部构成的三角形地带。由于在过去几年里印度各地恙虫病频繁爆发，该病被认为是一种复现传染病。本研究的目的是对2011年9月-2012年8月印度东北部一家医院的90例恙虫病病例的临床和辅助临床资料、并发症和疗效预测指标进行研究。

方法：2011年9月-2012年8月在印度梅加拉亚邦一家医院开展纵向研究。采用SD BIOLINE快速检测试剂盒（固相免疫层析试验）检测相关抗体（IgM, IgG or IgA）进行恙虫病诊断。对年龄、性别、地域、症状和体征,治疗方法、实验室结果、并发症,和预后结果进行描述性分析。计算多器官功能障碍综合征(MODS)和死亡的相对危险度(RR)及95%可信区间(*CI*)。通过二元逻辑回归的单变量显著性分析确认恙虫病MODS和死亡的预测指标。

结果：共对662例临床疑似恙虫病患者进行检测，最终确诊90例(13.6%)。90例中，男性占52.2%，平均年龄为36.29岁。发烧时间小于7 d(*n*=75, 83.3%)、干咳(*n*=21, 23.3%)、肝肿大(*n*=24, 26.7%)、脾肿大(*n*=22, 24.4%) 和淋巴腺病变 (*n*=20, 22.2%)是主要临床特征。10例(11.1%)观察到焦痂，至少有1/3的患者有一项全身性并发症。急性肝炎(*n*=15, 16.7%)、肺炎(*n*=14, 15.6%)和急性肾损伤(*n*=11, 12.2%)是主要并发症。14.4%(*n*=13)的病例具有MODS，并且38.5% (*n*=5)的患者因此而死亡。总体而言，恙虫病病死率为5.15% (*n*=5)。在单变量分析中，血小板计数<100 000/mm^3，血清肌酸酐>1.5 mg/dl和转氨酶>500 U/L及MODS ($P<0.001$)和死亡显著相关($P<0.05$)。另外，血清胆红素>3 mg/dl也与MODS ($P<0.001$)显著相关。当采用二元逻辑回归分析时，血清肌酸酐>1.5 mg/dl是MODS(*OR*: 76.1, 95%*CI*: 4.9-1 175.6)和死亡(*OR*: 18.03, 95%*CI*: 1.38-235.1)的主要预测指标。

结论：本研究中，约 1/7(13.6%)的急性未分化的发热由恙虫病引起。全身性并发症在恙虫病患者中非常普遍(33.3%)。血清肌酸酐>1.5 mg/dl 可作为 MODS 和死亡的预测指标。

Translated from English version into Chinese by Xin-Yu Feng, edited by Pin Yang

Profil clinique et paraclinique et facteurs prédictifs du résultat dans 90 cas de typhus des broussailles à Meghalaya (Inde)

Sunuraj Sivarajan, Siddharudha Shivalli, Debomallya Bhuyan, Michael Mawlong, Rittwick Barman

Résumé

Contexte: L’inde fait partie du « triangle à *tsutsugamushi* », une zone du monde dans laquelle le typhus des broussailles est endémique. Suite aux fréquentes épidémies récemment observées dans différentes régions du pays, on considère aujourd’hui que le typhus des broussailles est une maladie infectieuse réémergente en Inde. La présente étude visait à examiner le profil clinique et paraclinique, les complications et les facteurs prédictifs du résultat dans 90 cas de typhus des broussailles diagnostiqués dans un hôpital du nord-est de l’Inde entre septembre 2011 et août 2012.

Méthodes: Une étude longitudinale a été réalisée dans un hôpital de Meghalaya, en Inde, entre septembre 2011 et août 2012. Le typhus des broussailles a été diagnostiqué à l’aide du test rapide SD BIOLINE Tsutsugamushi (immunochromatographie en phase solide) qui recherche les anticorps IgM, IgG ou IgA. Une analyse descriptive des cas (âge, sexe, région géographique, symptômes et signes, traitements, résultats des analyses, complications et issue) a été réalisée. Le risque relatif (RR) avec un intervalle de confiance (*IC*) à 95 % a été calculé pour le syndrome de défaillance multiviscérale

(SDMV) et la mortalité. Une régression logistique binaire a été appliquée aux corrélats significatifs ($P < 0,05$) dans l'analyse univariée afin d'identifier les facteurs prédictifs du SDMV et de la mortalité dans le typhus des broussailles.

Résultats: Rien moins que 662 cas cliniquement suspects de typhus des broussailles ont été testés et 90 patients (13,6 %) ont été diagnostiqués positifs. Sur ces 90 patients, 52,2 % (*n* = 47) étaient des hommes et leur âge moyen était de 36,29 ans (ET 13,38). Les principales caractéristiques cliniques étaient de la fièvre pendant moins de 7 jours (*n* = 75, 83,3 %), des myalgies (*n* = 56, 62,2 %), des douleurs abdominales (*n* = 24, 26,7 %), des maux de tête (*n* = 24, 26,7 %), des nausées et vomissements (*n* = 21, 23,3 %), une toux sèche (*n* = 21, 23,3 %), une hépatomégalie (*n* = 24, 26,7 %), une splénomégalie (*n* = 22, 24,4 %) et des lymphadénopathies (*n* = 20, 22,2 %). Des escarres d'inoculation ont été observées sur 10 patients (11,1 %). Un tiers des patients (*n* = 30) ont développé au moins une complication systémique, principalement des hépatites aiguës (*n* = 15, 16,7 %), des pneumonies (*n* = 14, 15,6 %) et des lésions rénales aiguës (*n* = 11, 12,2 %). Un SDMV a été observé dans 14,4 % (*n* = 13) des cas et 38,5 % (*n* = 5) des patients atteints d'un SDMV sont décédés. Au total, la mortalité des cas a été de 5,15 % (*n* = 5). À l'analyse univariée, les plaquettes à < 100 000/mm^3, la créatinine sérique à > 1,5 mg/dl et les transaminases (ASAT, ALAT ou les deux) à > 500 U/l étaient associées au SDMV ($P < 0,001$) et à la mortalité ($P < 0,05$). En outre, un taux de bilirubine sérique > 3 mg/dl était également associé au SDMV ($P < 0,001$). Avec la régression logistique linéaire, un taux de créatinine sérique supérieur à > 1,5 mg/dl était prédictif du SDMV (*OR* : 76,1 ; *IC* à 95 % : 4,9-1175,6) et de la mortalité (*OR* : 18,03 ; *IC* à 95 % : 1,38-235,1).

Conclusion: Dans le cadre de la présente étude, un cas sur sept environ (13,6 %) de maladie fébrile aiguë indifférenciée était un typhus des broussailles. Les complications systémiques étaient fréquentes (33,3 %). Un taux de créatinine sérique supérieur à 1,5 mg/dl était prédictif du SDMV et de la mortalité.

Translated from English version into French by Suzanne Assenat, through

Клинический и параклинический профиль и прогностические параметры исхода для 90 случаев лихорадки цуцугамуши, Мегхалая, Индия

Sunuraj Sivarajan, Siddharudha Shivalli, Debomallya Bhuyan, Michael Mawlong, Rittwick Barman

Резюме

Общая информация. Индия входит в географический «треугольник *цуцугамуши*», где встречается это эндемическое заболевание. Из-за частых вспышек, происходивших в разных районах страны в недавнем прошлом, лихорадка цуцугамуши считается возвращающимся инфекционным заболеванием в Индии. Настоящее исследование посвящено изучению клинического и параклинического профиля, осложнений и предикторов исходам лихорадки цуцугамуши на основании 90 случаев этого заболевания, диагностированных в больнице северо-восточной Индии с сентября 2011 года по август 2012 года.

Методы. Продольное исследование проводилось в больнице города Мегхалая, Индия, с сентября 2011 года по август 2012 года. Диагностирование лихорадки цуцугамуши проводилось с помощью быстрого диагностического теста SD BIOLINE tsutsugamushi (методом твердофазного иммунохроматографического анализа) на наличие антител (IgM, IgG и IgA). Был проведен описательный анализ по возрасту, полу, географической области, симптомам и признакам, лечению, результатам лабораторных исследований, осложнениям и исходам. Относительный риск (ОР) с 95% доверительным интервалом (*ДИ*) был рассчитан для синдрома полиорганной дисфункции (СПОД) и смертности. Была применена бинарная логистическая регрессия к значимым корреляциям ($P < 0{,}05$) по даннным однофакторного анализа, с целью определения предикторов СПОД и смертности от лихорадки цуцугамуши.
Результаты. Было обследовано 662 пациента с клиническим подозрением на лихорадку цуцугамуши, из которых у 90 человек (13,6%) заболевание подтвердилось. Из 90 пациентов 52,2% (*n*= 47) были мужчины, и их средний (СО) возраст составлял 36,29 (13,38) лет. Основными клиническими проявлениями были: лихорадка <7 дней (*n*= 75, 83,3%), миалгия (*n*= 56, 62,2%), боли в животе (*n*= 24, 26,7%), головная боль (*n*= 24, 26,7%), тошнота/рвота (*n*= 21, 23,3%), сухой кашель (*n* = 21, 23,3%), гепатомегалия (*n*= 24, 26,7%), спленомегалия (*n*= 22, 24,4%) и увеличение лимфатических узлов (*n*= 20, 22,2%). Ожоговый струп был отмечен у 10 пациентов (11,1%). У одной трети пациентов (*n*= 30) развилось по крайней мере одно системное осложнение. В качестве общих осложнений наблюдались острый гепатит (*n*= 15, 16,7%), пневмония (*n*= 14, 15,6%) и острая почечная недостаточность (*n*= 11, 12,2%). СПОД был отмечен у 14,4% (*n*= 13), и 38,5% (*n*= 5) пациентов со СПОД умерло. В целом, коэффициент летальности составил 5,15% (*n*= 5). Однофакторный анализ показал, что уровни тромбоцитов <100 000/мм^3, креатинина сыворотки >1,5 мг/дл и трансаминазы (АСТ, АЛТ или оба) >500 ед./л связаны со СПОД (P<0,001) и смертностью (P<0,05). Кроме того, билирубин сыворотки >3 мг/дл также был связан со СПОД (P<0,001). С помощью бинарной логистической регрессии показано, что уровень креатинина сыворотки >1,5 мг/дл является предиктором СПОД (ИЛИ 76,1, 95%-й *ДИ*: 4,9-1 175,6) и смертности (*ИЛИ*: 18,03, 95%-й *ДИ*: 1,38-235,1).
Вывод. В условиях данного исследования примерно одна седьмая (13,6%) всех случаев острого недифференцированного лихорадочного заболевания была связана с лихорадкой цуцугамуши. Наблюдалось большое количество системных осложнений (33,3%). Уровень креатинина сыворотки >1,5 мг/дл являлся предиктором СПОД.

Translated from English version into Russian by Leila Usmanova, through

Perfil clínico y paraclínico e indicadores de resultados en 90 casos de tifus de las malezas, Meghalaya, India

Sunuraj Sivarajan, Siddharudha Shivalli, Debomallya Bhuyan, Michael Mawlong, Rittwick Barman

Resumen

Antecedentes: India es un componente integral del "triángulo *tsutsugamushi*" que representa una parte del planeta que es endémica para el tifus de las malezas. Debido a los frecuentes brotes que se han observado recientemente en distintas partes del país, en India se describe al tifus de las malezas como una enfermedad infecciosa re-emergente. El presente estudio tuvo como objetivo estudiar el perfil clínico y paraclínico, las complicaciones y los indicadores de resultado en 90 casos de tifus de las malezas diagnosticados en un hospital en el noreste de India entre septiembre del 2011 y agosto del 2012.

Métodos: Se llevó a cabo un estudio longitudinal en un hospital de Meghalaya, India entre septiembre de 2011 y agosto de 2011. Se llegó al diagnóstico de tifus de las malezas mediante prueba de diagnóstico rápida SD BIOLINE tsutsugamushi (ensayo inmunocromatográfico de fase sólida) para anticuerpos (IgM, IgG o IgA). Se llevaron a cabo análisis descriptivos de edad, género, zona geográfica, síntomas y signos, tratamiento, hallazgos de laboratorio, complicaciones y resultados. Se computó el riesgo relativo (RR) con intervalo de confianza (*IC*) de 95% para el Síndrome de Disfunción Multiorgánica (SDMO) y la mortalidad. Se aplicó regresión logística binaria a las correlaciones significativas (*P*<0.05) en análisis univariados para identificar los indicadores de SDMO y mortalidad en el tifus de las malezas.

Resultados: Se evaluaron 662 pacientes con sospecha clínica de tifus de las malezas y 90 (13,6%) fueron diagnosticados con tifus de las malezas. De los 90 pacientes, 52,2% (*n*=47) fueron masculinos y la edad promedio (SD) fue de 36,29 (13,38) años. Los signos clínicos predominantes fueron fiebre por <7 días (*n*=75, 83,3%), mialgia (*n*=56, 62,2%), dolor abdominal (*n*=24, 26,7%), dolor de cabeza (*n*=24, 26,7%), náuseas/vómitos (*n*=21, 23,3%), tos seca (n=21, 23,3%), hepatomegalia (*n*=24, 26,7%), esplenomegalia (*n*=22, 24,4%) y linfadenopatías (*n*=20, 22,2%). Se observaron escaras en 10 pacientes (11,1%). Un tercio (*n*=30) de los pacientes desarrolló por lo menos una complicación sistémica. Las complicaciones comunes fueron hepatitis aguda (*n*=15, 16,7%), neumonitis (*n*=14, 15,6%) y lesión renal aguda (*n*=11, 12,2%). Se observó SDMO en 14,4% (*n*=13) y 38,5% (*n*=5) de los pacientes con SDMO murieron. En general, el índice de mortalidad fue del 5,15% (*n*=5). En el análisis univariado, las plaquetas <100 000/mm^3, la creatinina sérica >1.5 mg/dl y la transaminasa (AST, ALT o ambas) >500 U/L estuvieron asociadas con SDMO (*P*<0,001) y mortalidad (*P*<0,05). Además, también se observó una relación entre la bilirrubina sérica >3 mg/dl y SDMO (*P*<0,001). Al aplicar la regresión logística binaria, la creatinina sérica >1.5 mg/dl fue indicador de SDMO (*RM*: 76,1, 95% *IC*: 4.9-1 175,6) y mortalidad (*RM*: 18,03, 95% *IC*: 1.38-235,1).

Conclusión: En el marco de este estudio, aproximadamente una séptima parte (13,6%) de la enfermedad febril aguda indiferenciada fue debido a tifus de las malezas. Las complicaciones sistémicas fueron comunes (33,3%). La creatinina sérica >1.5 mg/dl fue indicador de SDMO y mortalidad.

Translated from English version into Spanish by Maria Alejandra Aguada, through

TRANSLATORS
WITHOUT BORDERS
