# Supplementary material for: Clinical and paraclinical profile, and predictors of outcome in 90 cases of scrub typhus, Meghalaya, India
Source: Infect Dis Poverty. 2016 Oct 5;5:91. doi: 10.1186/s40249-016-0186-x (PMC5051022; doi:10.1186/s40249-016-0186-x)
Supplement: Additional file 2: — Findings of liver function test among patients with scrub typhus and serum bilirubin >3 mg/dl in a tertiary care hospital of Meghalaya, India, Sept 2011–Aug 2012 (n = 11). (DOCX 13 kb) [file 40249_2016_186_MOESM2_ESM.docx]

**Findings of liver function test among patients with scrub typhus and serum bilirubin >3mg/dl in a tertiary care hospital of Meghalaya, India, Sept 2011-Aug 2012 (n=11)**

| **No** | **S.bilirubin (mg/dl)** | | | **AST^†^ (IU/L)** | **ALT^††^ (IU/L)** | **ALP^§^ (IU/L)** |
| --- | --- | --- | --- | --- | --- | --- |
|  | Total | Direct | Indirect |  |  |  |
| 1 | 4 | 3.1 | 0.9 | 850 | 323 | 292 |
| 2 | 4 | 3.3 | 0.70 | 423 | 76 | 676 |
| 3 | 4.4 | 3.90 | 0.50 | 161 | 96 | 461 |
| 4 | 4.6 | 3.70 | 0.90 | 153 | 102 | 503 |
| 5 | 5.6 | 2.70 | 2.90 | 230 | 97 | 90 |
| 6 | 5.8 | 5.5 | 0.3 | 460 | 60 | 145 |
| 7 | 5.8 | 5.5 | 0.30 | 470 | 60 | 531 |
| 8 | 10.2 | 9 | 1.2 | 184 | 113 | 1030 |
| 9 | 10.4 | 1.1 | 9.3 | 623 | 103 | 450 |
| 10 | 11.5 | 9.2 | 1.7 | 654 | 140 | 300 |
| 11 | 11.5 | 10.4 | 1.1 | 631 | 106 | 630 |

**^†^**AST: Aspartate aminotransferase; **^††^**ALT: Alanine aminotransferase ; **^§^**ALP: Alkaline phosphatase
